# Inhibition of calpain9 attenuates peritoneal dialysis-related peritoneal fibrosis

**DOI:** 10.3389/fphar.2022.962770

**Published:** 2022-12-01

**Authors:** Fang Li, Yu Wang, Jianwei Tian, Zhanmei Zhou, Wei Yin, Xianhui Qin, Huizhen Wang, Tao Zeng, Aiqing Li, Jianping Jiang

**Affiliations:** ^1^ Division of Nephrology, Nanfang Hospital, Southern Medical University, National Clinical Research Center for Kidney Disease, State Key Laboratory of Organ Failure Research, Guangdong Provincial Key Laboratory of Renal Failure Research and Guangdong Provincial Clinical Research Center for Kidney Disease, Guangzhou, China; ^2^ Laboratory Animal Research Center of Nanfang Hospital, Southern Medical University, Guangzhou, China

**Keywords:** calpain, calpain 9, fibrosis, peritoneal dialysis, peritoneal mesothelial cells

## Abstract

**Aim:** Peritoneal dialysis is a common renal replacement method for end-stage renal disease. Long-term peritoneal dialysis leads to peritoneal dialysis-related peritoneal fibrosis, which leads to a cessation of treatment. Calpain is a protein belonging to calcium-dependent endopeptidase family and plays an important role in extracellular matrix remodeling. Here, we evaluated the effect of calpain in peritoneal dialysis-related peritoneal fibrosis.

**Methods:** We established two animal models of peritoneal fibrosis and inhibited the activity of Calpain, and then collected peritoneal tissue to evaluate the progress of fibrosis and the changes of Calpain and β-catenin. We obtained Rat peritoneal mesothelial cells and Human peritoneal mesothelial cell line and stimulated with TGF-β to produce extracellular matrix. Next we inhibited Calpain activity or reduced Calpain9 expression, and then assessed changes in extracellular matrix and β-catenin.

**Results:** Inhibition of calpain activity attenuated chlorhexidine glucose and peritoneal dialysis-induced peritoneal thickening and β-catenin expression in mice. In addition, compared with the control group, when primary rat peritoneal mesothelial cells or human peritoneal mesothelial cells were treated with transforming growth factor beta, down-regulation of calpain activity inhibited the expression of Fibronectin and Collagen I, and increased the expression of E-cadherin. These changes could be adjusted after silencing calpain9. Finally, calpain9 deficiency was associated with down-regulation of Fibronectin and β-catenin in human peritoneal mesothelial cells.

**Conclusion:** Our results suggest that calpain9 may be a key molecule in mediating peritoneal dialysis-related peritoneal fibrosis.

## Introduction

According to reports in 2020, the United States, Japan and China are the high incidence areas of end-stage renal disease (ESRD). (Yang et al., 2020; Johansen et al., 2021).Peritoneal dialysis (PD) is one of the recognized renal replacement therapies. However, about 50% of patients who received continuous ambulatory peritoneal dialysis (CAPD) for more than 6 years had excessive capacity load and ultrafiltration failure. (Ditsawanon and Aramwit, 2015).Over time, PD patients are involved in the decline of renal function and the occurrence of peritoneal fibrosis, which eventually leads to the failure of peritoneal dialysis treatment. (Jagirdar et al., 2019).

During PD treatment, nonbiocompatible dialysis fluid (high concentration of glucose, low pH, hypertonic, glucose degradation products, and advanced glycation end-products), peritonitis, uremia and other factors, initiated the activation of peritoneal innate cells, secretion of a variety of cytokines, promoting the development of fibrosis. (Zhou et al., 2016; Roumeliotis et al., 2020). Therefore, the prevention of peritoneal fibrosis in PD patients is one of the focuses of PD treatment. (Balzer, 2020). At present, a high concentration of glucose is still used as an osmotic medium for liquid ultrafiltration, which is widely used in PD solutions. Continuous exposure to non-physiological high concentration of glucose can lead to peritoneal injury and eventually lead to peritoneal fibrosis. (Grantham et al., 2017). The current treatment of peritoneal fibrosis is limited, although in recent years, higher biocompatible solutions have made significant progress. However, no research or meta-analysis showed that the use of biocompatible solutions differed in terms of technical failure or patient survival. (Blake, 2018). Other drugs have been proved in experimental studies, but most have not been tested in further clinical trials. Anti-inflammatory and anti-fibrotic agents targeting cell pro-inflammatory factors and pro-fibrotic factors may play a role in protecting the peritoneum, but their specific usage and risks have not been further improved. (Auxiliadora Bajo et al., 2017).Therefore, we should explore new peritoneal fibrosis targets and anti-fibrosis strategies.

Epithelial to mesenchymal transformation (EMT) of peritoneal mesenchymal cells is considered to be the early process of peritoneal fibrosis. The process of EMT mainly consists of the gradual disappearance of the epithelial phenotype of peritoneal mesothelial cells accompanied by an increase in fibrocytoid features, which are characterized by cytoskeletal rearrangement, the acquisition of migration and invasion capabilities, and extracellular matrix (ECM) deposition.

Calpain is a calcium-dependent cysteine protease distributed in the cytoplasm. There are 15 isoenzymes. Calpain1 and calpain2 are two typical representatives of calpain, respectively. Calpain1 and calpain2 are a large 80 kD catalytic subunit and a common 30 kD small subunit (CAS1) to help maintain the activity of calpain. And it is reported that calpain9 is mainly expressed in the gastrointestinal tract, while calpainS2 is mainly expressed in the skin and esophagus. (Ono et al., 2016; Spinozzi et al., 2021).It is believed to be related to cytoskeleton remodeling during cell fusion and exercise, molecular hydrolysis modification in signal transduction pathway, enzyme degradation regulating cell cycle, gene expression regulation, and substrate degradation in some apoptotic pathways. Calpain is involved in many cellular functions in a calcium-dependent manner and has a variety of protein substrates. (Goll et al., 2003).However, the exact physiological functions of calpain are still unclear. With the discovery and further understanding of some new members of the calpain family, it is suggested that calpain may be involved in certain functions other than proteolysis. (Arnandis et al., 2012). In recent years, calpain has been found to play an important role in mesothelial fibrosis. (Schwenger et al., 2006; del Peso et al., 2016). The activation of calpain can induce Collagen I synthesis and cell proliferation of mesothelial cells. (Yang et al., 2016). Recently, gastrointestinal calpain9 of the calpain family has been reported as a therapeutic target for alleviating fibrosis induced by transforming growth factor β (TGF-β) in mouse breast cancer cells, human lung fibroblasts, canine kidney cells and human vascular endothelial cells. (Kim et al., 2019).

Although recent reports have shown that calpain can promote fibrotic disorders, (Letavernier et al., 2012), the role of calpain in peritoneal dialysis induced by long-term PD is still poorly understood. This study investigated the activation of calpain and the role of calpain9 in peritoneal dialysis-related peritoneal fibrosis. After treatment with peritoneal dialysis fluid (PDF) and chlorhexidine glucose (CG), the activity of calpain was increased and the fibrosis was aggravated. Similarly, TGF-β-induced extracellular matrix (ECM) in Rat peritoneal mesothelial cells (RPMC) and Human peritoneal mesothelial cell line (HmrSV5) was increased and E-cadherin expression was increased when calpain activity was down-regulated or calpain9 is deficient. In addition, we verified that the accumulation of β-catenin and snail in the cells was reduced when calpain9 was knocked down, which may suggest that calpain9 regulates peritoneal fibrosis.

## Materials and methods

### Animal

CD-1 mice weighing 25–30 g were purchased from the Experimental Animal Center of Southern Medical University (Guangzhou, China). For peritoneal fibrosis model, the mice were injected with 0.1%chlorhexidine gluconate and 15% ethanol (CG, 0.3 ml 25 g body wt^−1^, i. p. daily) for 21 days (Ishii et al., 2001; Si et al., 2019). In addition, we injected 4.25% peritoneal dialysis fluid (PDF, 100 ml kg body wt^−1^, i. p. daily) into the peritoneal cavity of mice daily for 6 weeks to establish another model of peritoneal fibrosis (Peng et al., 2011). The control (Ctrl) group mice were treated with an equal volume of 0.9% saline.

MDL28170 is a widely used broad-spectrum calpain inhibitor, and it does not affect TGF-β phosphorylation or nuclear accumulation. (Ono et al., 2016; Kim et al., 2019).To explore the therapeutic treatment of Calpain on peritoneal fibrosis, MDL28170 was designed to treatment by gavage with 100 mg kg body wt^−1^ at day 11 after CG injection and then delivered twice daily (Pollock, 2005; Kim et al., 2019). All mice were assigned to four groups, and six mice in each group: the sham group was given an equal volume of 0.9% saline; the MDL28170 group was given MDL28170 by oral gavage without the CG injection; the CG group administered CG 300 μl 25 g body wt^−1^ daily for 2 days; the CG + MDL28170 group was treated with CG and then given MDL 28170 at day 11 as previously described (Kim et al., 2019). As for the PDF group, the group of mice in this group was the same as the CG group. The mice were orally fed with MDL28170 on the first day of week four in the same way. The mice in the PDF group were sacrificed at the sixth week. The anterior abdominal peritoneum was collected for immunostaining. The visceral peritoneum was collected for western blotting. The animal study was reviewed and approved by Institutional Animal Care and Use Committee at Nanfang Hospital.

### Rat peritoneal mesothelium cell isolation and confirmation

We choose to separate the omentum tissue of male SD rats (200–250 g), mix it with preheated 0.25% trypsin (Amresco, United States) and 0.02% EDTA, and then shake and digest at 37°C. After digestion, the tissue pieces were removed, the cell suspension was centrifuged at 80xg for 10 min at 4°C, and then supplemented with 10% fetal bovine serum (FBS, Gibco, United States), penicillin (100 IU mL^−1^, Gibco, United States) and streptomycin (100 mg mL^−1^, Gibco, United States) were cultured in Dulbecco’s Modified Eagles Medium (DMEM)/F12 medium at 37°C and 5% CO_2_. Replace media of fresh complete medium every 2 days until 70%–80% confluency. The mesothelial cell surface markers, including vimentin and cytokeratin 18, were confirmed by immunofluorescence. Deletion of Calpain9 in mesothelial cells reduces ECM protein expression.

### Cell culture and treatments

RPMC and HMrSV5 were cultured in DMEM supplemented with 10% fetal bovine serum, 1% penicillin, and streptomycin in an atmosphere at 37°C and 5% CO_2_. To determine the effect of calpain9 on RPMC in response to TGF-β. RPMC were treated with TGF-β at 10 ng mL^−1^ for 48 h or pretreated with MDL28170 at 10, 20, and 30 μM for 1 h HMrSV5 cells were transfected with siRNA knockdown of calpain9 or negative control (GenePharma, China) before TGF-β incubation. The cells were collected for Western blot analysis and immunofluorescence staining.

### Transfection

According to the manufacturer’s instructions, Lipofectamine RNAiMAX reagent (Life Technologies) and siRNA against calpain9 or Negative Control were added to the medium and the cells were incubated for 20 min. New serum-free medium is added to the cells, and the mixed siRNA reagent is added to make the final concentration of siRNA 100 nM. After 6 h of incubation, the medium was changed and TGF-β is added as described previously.

### Western blot

Western blot was used to detect the activation state of calpain9, the expression of FN and Col-1 in peritoneal tissues and RPMC. In order to measure whether calpain is activated in peritoneal and peritoneal mesothelial cells, an antibody that specifically recognizes SBDP (MERCK Millipore, United States) was used to determine the degradation products of αII spectrin. αII spectrin contains a specific calpain cleavage site, and calpain can decompose the full length of 250 kD SBDP into 150 kD and 120 kD products. This method has been widely used to detect calpain activation. (Saido et al., 1993).

Peritoneal tissue and RPMC are lysed in RIPA lysate and protease inhibitors. Using BCA protein quantification kit (Beibo, China) for protein quantification. The same amount of total protein (20 μg) was separated by SDS/PAGE electrophoresis, 5% skimmed milk powder was blocked for 1 h and then transferred to nitrocellulose membrane, and anti-rat β-actin (Proteintech, United States), GAPDH(Proteintech, United States), Fibronectin (FN) (Proteintech, United States), Collagen-I (Col-I) (Boster, China), α-Spectrin (SBDP) (Millipore,United States), calpain9 (Proteintech, United States), active β-catenin (Cell Signaling Technology,United States), E-Cadherin (Cell Signaling Technology,United States), p Smad2/3(Cell Signaling Technology,United States), Smad2/3 (Affinity, China), Snail (Abcam, United Kingdom) primary antibodies, incubated overnight at 4°C, the next day after washing the membrane with TBST, incubate with the corresponding fluorescent secondary antibody (LICOR, United States) for 1 h at room temperature. Images were collected in the gel imaging and chemiluminescence image analysis system (Odyssey, United States). ImageJ software was used for quantitative analysis of western blotting.

### Immunofluorescence staining

The cells of different groups were evenly spread on cover slips in a six-well plate and cultured to confluence. The cells were washed with PBST, fixed with methanol at −20°C for 20 min, and then wash with PBST and block with 5% BSA for 1 h and then combined with α-SMA (Proteintech, United States), cytokeratin 18 (Proteintech, United States), Vimentin (Proteintech, United States) and calpain9 (Proteintech, United States) primary antibody overnight at 4°C then washed with PBST and then incubated with fluorescent secondary antibody at room temperature for 1 h. The nuclei were counterstained with diamidinophenylindole (DAPI) (Beijing Zhongshan Jinqiao, China), and the images were observed and collected with a fluorescent inverted microscope (OLYMPUS, Japan).

### Statistics

Data were shown as mean ± SEM, and SPSS 18.0 statistical software was used. One-way analysis of variance with LSD multiple-comparison test or nonparametric Mann–Whitney U test were used for statistics between groups, and *p*-value < 0.05 was considered significant.

## Results

### Calpain activity and calpain9 expression are increased during peritoneal fibrosis

**FIGURE 1:**
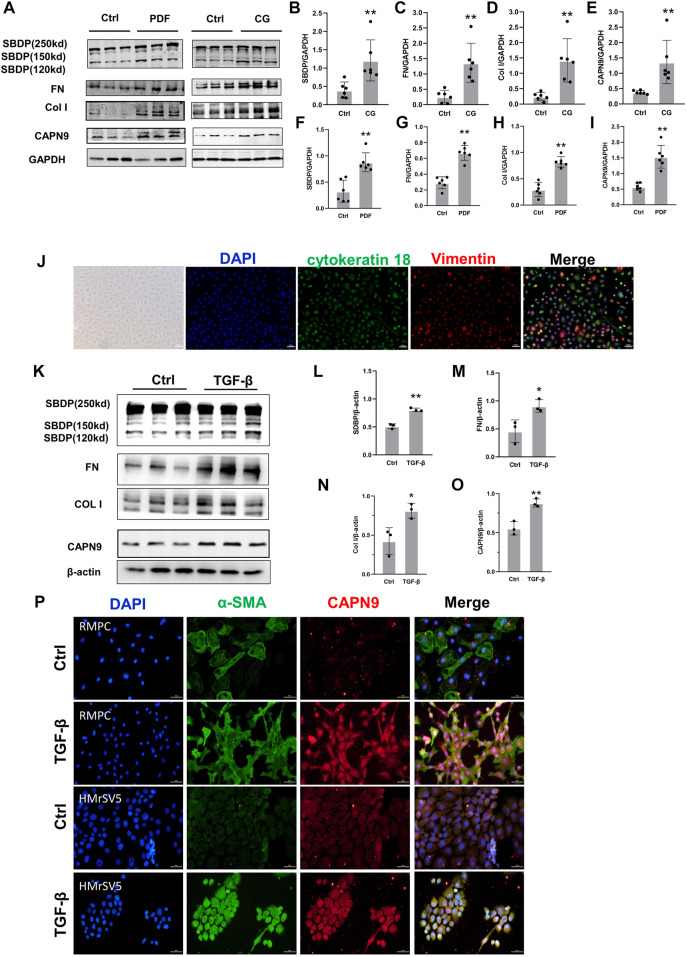
Calpain activity and calpain nine expression is increased during peritoneal fibrosis. **(A)**. Western blotting (WB) showing the expression of Calpain9 (CAPN9), SBDP cleavage products (120 and 150 kD), Collagen I (Col I) and Fibronectin (FN) in visceral peritoneal lysates of untreated (Ctrl), 6-week PDF **(F–I)** or 21-day CG treated mice. **(B–E)** SBDP (120 and 150kD), FN, Col I, CAPN9 in the peritoneal were examined. GAPDH was detected as loading control. Values are the mean ± s. e.m. *n* = 6, *p < 0.05, **p < 0.01, vs. control (Ctrl). **(J)** Isolation and identification of RPMC. Immunofluorescence staining was used to identify the isolated RPMC. Vimentin was labeled with red, cytokeratin 18 was stained with green, and the nucleus was stained with blue. Magnification objective, ×20. Scale bar: 50 μm. **(K–O)** Western blot was used to detect the expression of SBDP (120 and 150kD), FN, Col I, CAPN9 in total lysates of RPMC untreated (Ctrl) or treated with 10 ng/ml TGF-β1 for 48 h β-actin was detected as a control. Values are the mean ± s. e.m. *n* = 3, *p < 0.05,**p < 0.01, vs. control (Ctrl). **(P)** Increased expression of CAPN9 induced by TGF-β in RPMC(top) and HmrSV5 (bottom). The cells were fixed, permeable and closed and stained with monoclonal antibodies against α-SMA and CAPN9. Immunofluorescence inverted microscope showed that green represented α-SMA, and red represented CAPN9. Magnification objective, × 20. Scale bar: 50 μm.

We found that the activity of calpain increased during peritoneal fibrosis. The cleavage product of SBDP proved to be an early biomarker for pulmonary fibrosis induced by tuberculous pleurisy (Hong et al., 2018). In the dialysis model group, the cleavage products of specific substrates of calpain were higher than those in the control group, indicating that calpain in the model group was activated. At the same time, the expression level of calpain9 in the dialysis model group was higher than that in the control group (Figures 1A–I). *In vitro*, we Isolated RPMC and identified it by immunofluorescence using antibodies against Vimentin and cytokeratin 18 (Figure 1J). When RPMC and HMrSV5 were stimulated by TGF-β, the extracellular matrix was deposited, and both the activity of calpain and the expression of calpain9 were increased (Figures 1K–P), which suggests that calpain may be involved in the development of peritoneal dialysis-related peritoneal fibrosis in rats.

### Calpain inhibition attenuates development of peritoneal fibrosis in the peritoneum

**FIGURE 2:**
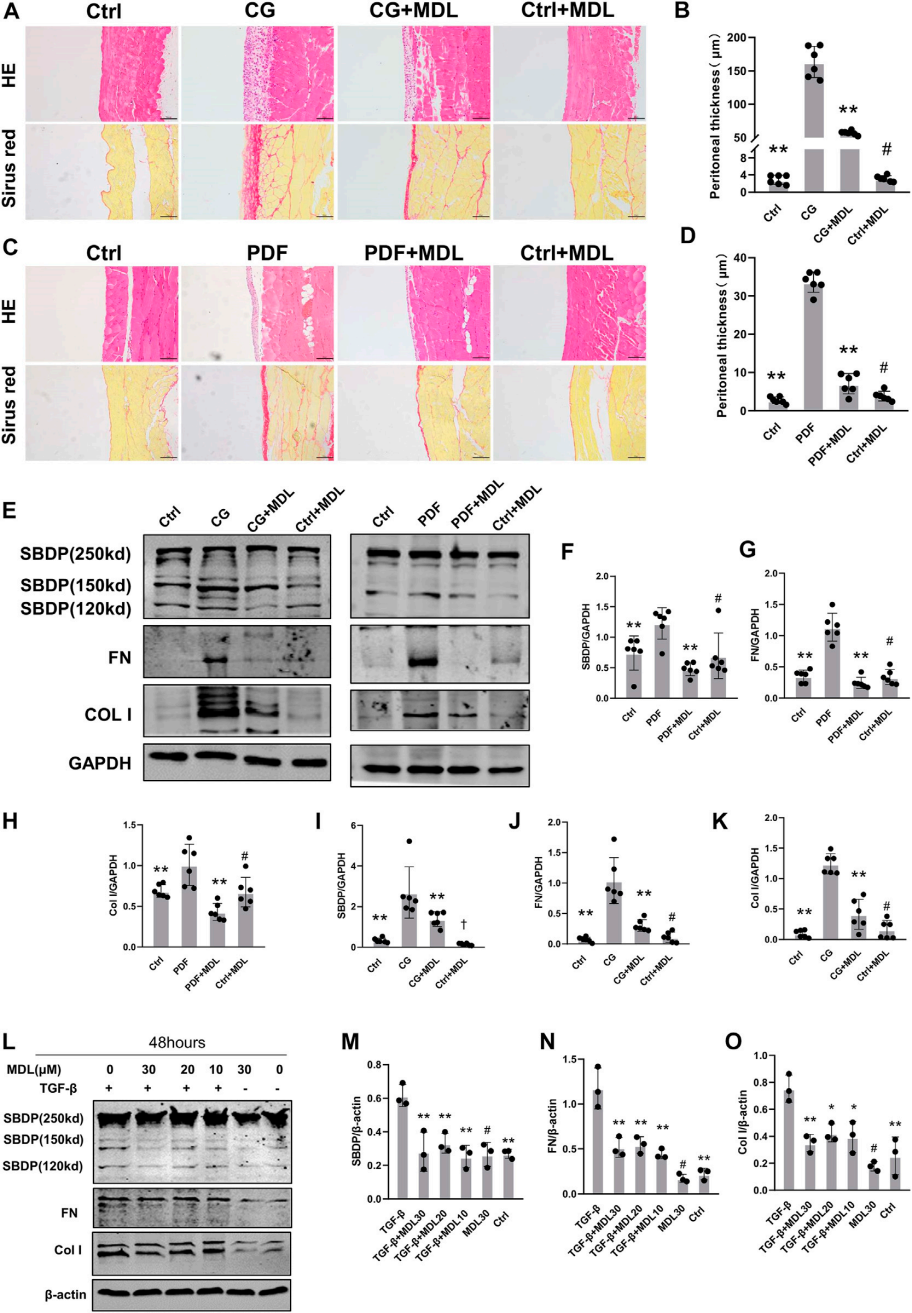
Inhibition of calpain activity can prevent the occurrence and progression of peritoneal fibrosis. **(A,B)** Peritoneal collection 21 days after CG injury after receiving or not receiving MDL (MDL28170) treatment (100 mg/kg/d). The HE and Sirius red staining of the peritoneal were observed by microscope, and the thickness of the dense area was measured (magnification, × 200, scale 100 μm). **(C,D)** Peritoneal collection 6 weeks after receiving or not receiving MDL (MDL28170) (100 mg/kg/d), 4.25% PDF intraperitoneal infusion. Values are the mean ± s. e.m. *n* = 6, *p < 0.05, **p < 0.01, vs. CG or PDF group. #*p* > 0.05. vs. control (Ctrl). **(E–K)** Western blot showing the expression of FN, Col I, and SBDP cleavage products (150kD), in total lysates of 2 models with or without MDL (MDL28170). GAPDH was detected as a loading control. Values are the mean ± s. e.m. *n* = 6, *p < 0.05, **p < 0.01, vs. CG or PDF group. #*p* > 0.05. vs control (Ctrl). ^†^ *p* < 0.01, vs. control (Ctrl). **(L–O)**. After TGF-β stimulation, the presence or absence of MDL (MDL28170) in RPMC was analyzed by Western blotting to detect ECM proteins. β-actin was detected as a loading control. Values are the mean ± s.e.m. *n* = 3, *p < 0.05, **p < 0.01, vs. TGF-β group. #*p* > 0.05. vs. control (Ctrl).

In order to detect whether calpain is associated with peritoneal fibrosis, we chose to orally feed calpain inhibitor MDL28170 (100 mg kg-1) twice a day after intraperitoneal injection of CG for 11 days. By means of pathological staining, we observed that the peritoneum of mice orally administered with MDL28170 after intraperitoneal injection of CG was thinner than that of mice injected with CG alone (Figure 2A). There was no thickening of peritoneal tissue in control group and mice fed MDL28170 alone. We detected changes of several proteins in each group of mice by immunoblotting. As shown in the figure, after blockade of calpain activity by MDL28170, the cleavage products of SBDP decreased, and the expression of FN and Col I induced by CG also decreased (Figure 2C). We also applied a high glucose concentration peritoneal dialysis model, as shown in Figure 2, which was also divided into four groups. Similarly, we found that the membrane thickness of mice receiving peritoneal dialysis solution and MDL28170 group was lower than that of mice receiving peritoneal dialysis solution alone (Figure 2B). Western blot also showed that the expression levels of FN and Col I induced by high glucose decreased after inhibiting calpain activity (Figure 2C). Our two models show that calpain activation is closely related to the development of peritoneal fibrosis.

To explore the role of calpain in TGF-β-induced type I collagen synthesis and extracellular matrix deposition, RPMC was incubated with TGF-β in the absence or presence of MDL28170. We found that MDL28170 inhibited TGF-β-induced increase in calpain activity and type I collagen synthesis in RPMC (Figure 2D). These results further support the involvement of calpain in collagen I synthesis and extracellular matrix deposition.

### Deletion of Calpain9 in mesothelial cells reduces ECM protein expression

**FIGURE 3:**
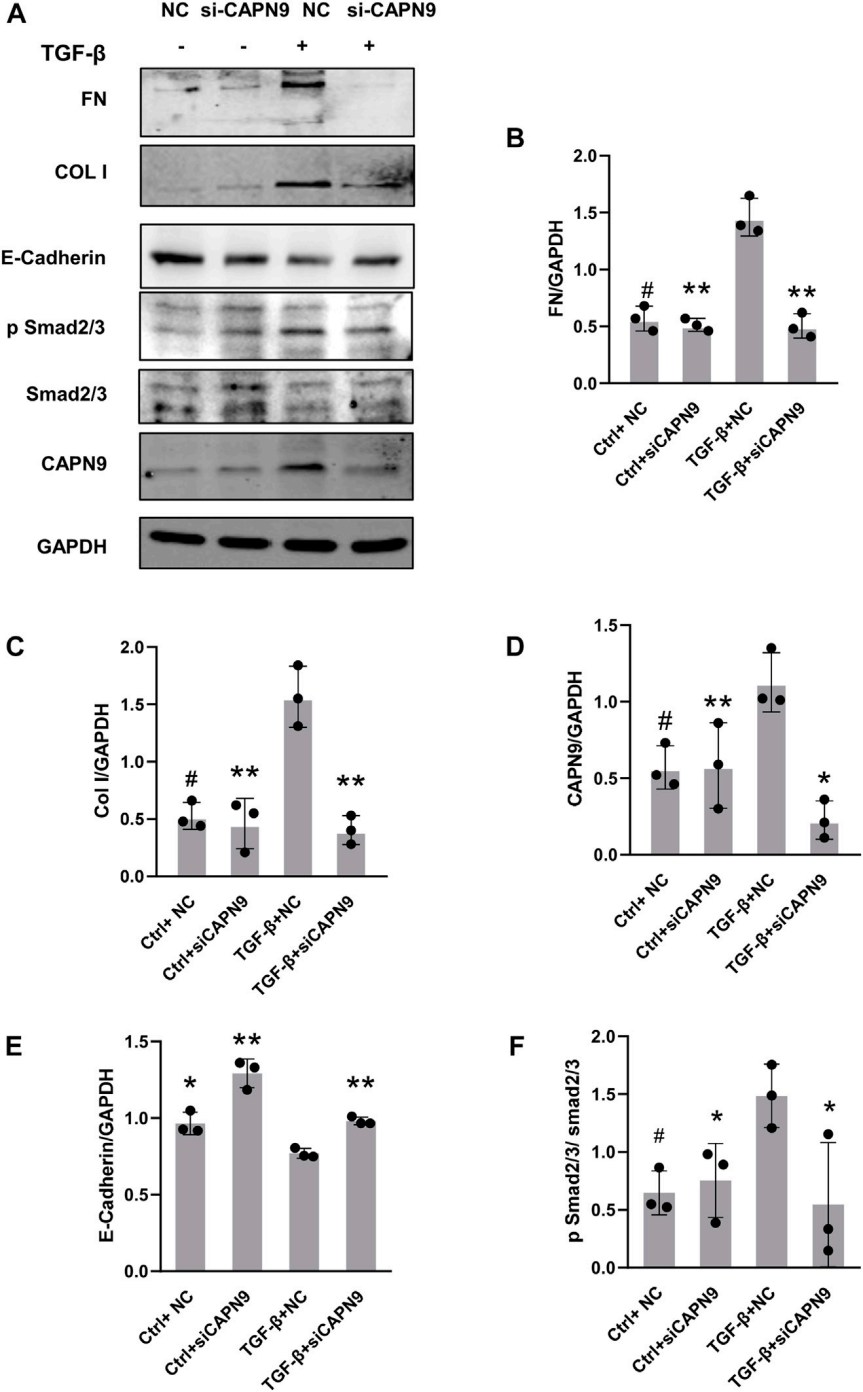
Calpain9 deficiency attenuated TGF-β-induced ECM protein expression and EMT. **(A–F)** Western blot showed Fibronectin (FN), Collagen I (Col I), Calpain9 (CAPN9), E-Cadherin, p Smad2/3 and Smad2/3 expression in whole cell lysates with or without TGF-β, HmrSV5, NC control or CAPN9-targeted siRNA transfection. *p < 0.05, **p < 0.01, vs. TGF-β group.#*p* > 0.05. vs negative control (NC).

Current calpain inhibitors are still not sufficiently specific to distinguish calpains from other proteases. So we selectively knocked down calpain9. After transfection of calpain9 siRNA into HMrSV5, we examined the changes of phosphorylated Smad2/3, a downstream signaling molecule of TGF-β. After knockdown of Calpain9 (CAPN9), the expression of phosphorylated Smad2/3 decreased. We also found that compared with the vector treatment, the protein expressions of Col I and FN were significantly reduced after the knock-down of calpain9 by siRNA. In addition, we detected the production of E-cadherin, the markers of epithelial cell marker (Figure 3). We also detected the expression of αSMA, but we found no significant difference between the groups (Supplementary Figure S1).

### MDL28170 inhibits calpain activity and calpain9 knockdown inhibits activation of β-catenin signaling pathway in peritoneal fibrosis

**FIGURE 4:**
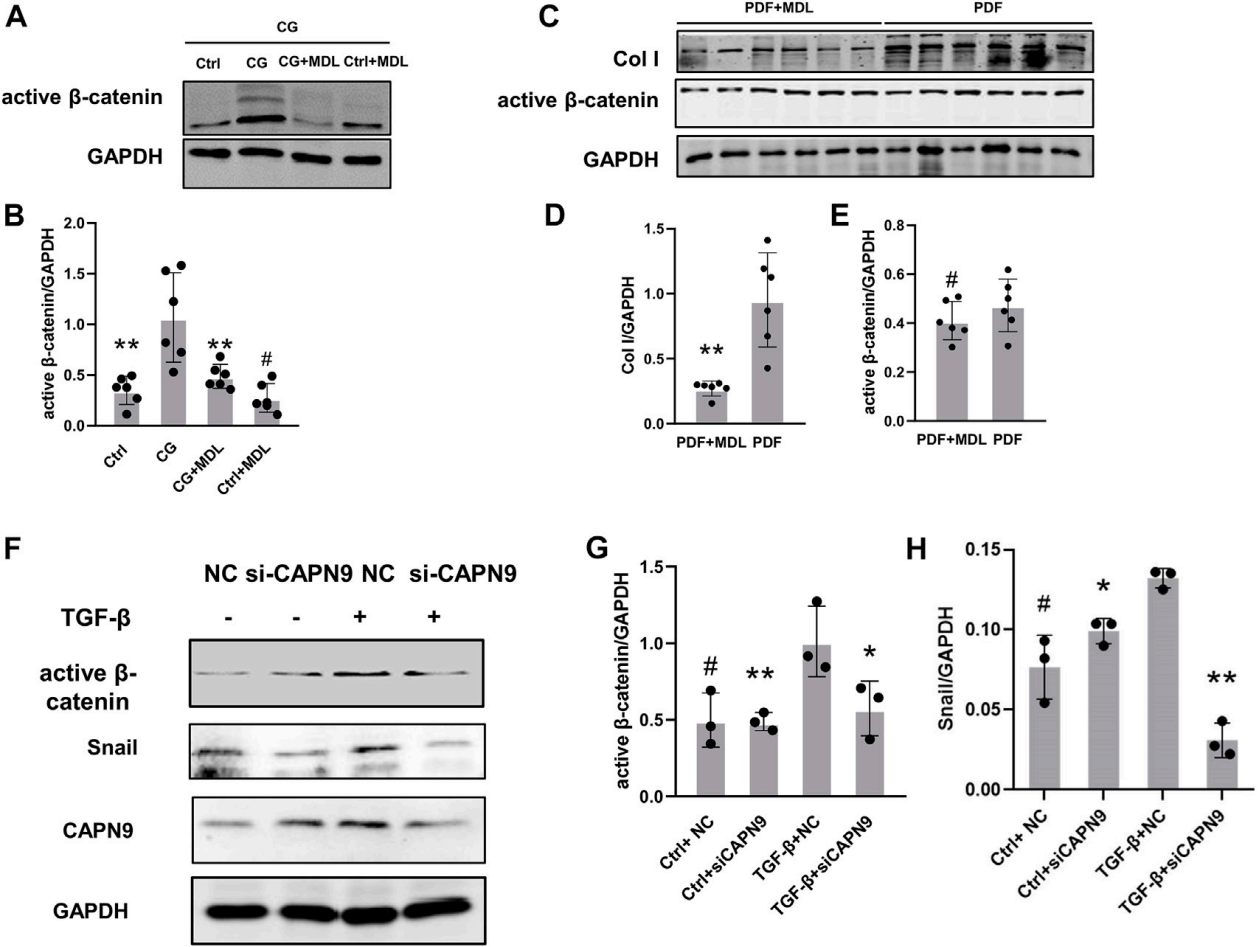
Chlorhexidine glucose treatment elevated the expression of active β-catenin in peritoneum and knockdown of Calpain9 resulted in down-regulation of active β-catenin. **(A–E)**. Western blotting showed the expression of active β-catenin, snail and extracellular matrix proteins in peritoneal lysates of mice in chlorhexidine glucose (CG) group **(A,B)** and peritoneal dialysis fluid (PDF) group **(C–E)**. *n* = 6 per group. *p < 0.05, **p < 0.01, vs. CG or PDF group. #*p* > 0.05. vs control (Ctrl). **(F–H)**. The expression of active β-catenin and snail was detected with or without siRNA-CAPN9 after TGFβ stimulation. *p < 0.05,**p < 0.01, vs. TGF-β group.#*p* > 0.05. vs. negative control (NC).

In order to further explore the mechanisms of peritoneal fibrosis, we detected the activation of β-catenin in CG and peritoneal dialysis fluid models. We also examined the changes of active β-catenin and snail after inhibition of calpain activity and knockdown of calpain9. After HMrSV5 cells were treated with TGF-β, we did not find significant changes of TGF-β levels in the cell supernatant after CAPN9 knockdown in the presence or absence of TGF-β (Supplementary Figure S2). Then we found nuclear translocation of β-catenin in the cells (Supplementary Figure S3), indicating that β-catenin activation was induced. And our results suggest that active β-catenin and snail may increase in response to CG and TGF-β stimulation, MDL28170 treatment and calpain9 knockdown inhibit FN and Col I expression and significantly prevent the increase of active β-catenin and snail (Figures 4A–H). However, in the peritoneal dialysis fluid model, the change of β-catenin was not obvious after MDL28170 treatment (Figures 4A,B).

## Discussion

PD-related peritoneal fibrosis is the main cause of peritoneal failure. Exploring the mechanisms of the occurrence and development is of great significance for the prevention and treatment of PD-related peritoneal fibrosis. In this study, the results show that the activation of calpain is involved in the process of PD-related peritoneal fibrosis and plays a role in promoting peritoneal fibrosis, and inhibiting the activity of calpain can alleviate the degree of peritoneal fibrosis. Calpain9 can be induced by TGF-β, and knocking down calpain9 can reduce the expression of ECM components.

Calpain is also considered as a key molecule in organ or tissue reconstruction. In the calpain1 knockout mouse model of pulmonary fibrosis, pulmonary hypertension-induced vascular remodeling was significantly improved. (Ma et al., 2011). The inhibition of calpain can improve pulmonary fibrosis induced by angiotensin II, collagen overexpression and fibrosis of pleural mesothelial cells. (Tabata et al., 2010). Cleavage products of SBDP were shown to be independent predictors of pleural fibrosis induced by tuberculous pleurisy. In tuberculous pleurisy, high pleural levels of SBDP breakdown products may indicate the presence of pleural fibrosis (Hong et al., 2018). In tuberculous pleurisy, high pleural levels of SBDP breakdown products may indicate the presence of pleural fibrosis (Hong et al., 2018).

In this study, we used two peritoneal dialysis disease models, representing peritoneal fibrosis and sclerosing encapsulated peritonitis caused by long-term peritoneal dialysis, respectively (Alshomimi et al., 2021). By intraperitoneal injection of peritoneal dialysis solution for 6 weeks or intraperitoneal injection of CG for 21 days, the mice were sacrificed, and the peritoneal tissue of PDF or CG mice was thickened and showed fibrosis pathological changes; the activity of calpain in tissues increased, and the expression of calpain9 was up-regulated. Pathological images of mice in each group showed that peritoneal thickening and collagen fiber hyperplasia were obvious in peritoneal dialysis group. MDL28170, a calpain inhibitor that can penetrate the cell membrane, was selected as to block calpain s activation in this study (Ma et al., 2011; Kim et al., 2019; Sun et al., 2020). Nonselective inhibitor of calpain MDL28170 treatment can significantly inhibit a series of changes in peritoneal tissue. This indicates that the activation of calpain can aggravate peritoneal fibrosis *in vivo*.

The key factor involved in peritoneal dialysis-related peritoneal fibrosis is TGF-β. The activation of TGF-β is an important early event of peritoneal fibrosis. In the context of the wide application of biocompatible peritoneal dialysis solutions, high concentrations of glucose, glucose degradation products (GDP) and advanced glycation end products (AGEs) can induce the production of TGF-β and promote the occurrence and development of peritoneal fibrosis. And previous studies have reported that TGF-β1 is the potential substrate of calpain. When exogenous calpain is added to the medium, calpain activates TGF-β1 by lysing the incubation period-related peptide (LAP) on the surface of endothelial cells. (Abe et al., 1998; Ma et al., 2011).

In this study, through molecular biological detection, it was found that TGF-β induced the increase of calpain activity and calpain9 expression level in peritoneal mesothelial cells. After pretreatment with calpain inhibitor MDL28170 or silencing calpain9, it was found that TGF-β-induced changes in RPMC fibrosis markers were inhibited.

Previous studies on calpain have shown that calpain plays a further role in the activation of TGF-β and the up-regulation of downstream related gene transcription. TGF-β1 induced a rapid, transient and significant increase in intracellular Ca^2+^ concentration *in vitro*. (Yoshimura et al., 1983; Moretti et al., 2014). After the intracellular calcium concentration increases, the activated calpain can release TGF-β from the dormant complex, and TGF-β regulates the transcription of downstream related fibrosis molecules through Smad 2/Smad 3/CTGF pathway. (Inomata et al., 1984; Imajoh et al., 1986). It suggesting that calpain plays an important role in mediating TGF-β-induced fibrosis. After we knocked down CAPN9, the expression of phosphorylated Smad2/3 decreased. This indicates that capn9 is related to Smad-dependent TGF-β transduction pathway.

We also further explored how calpain participates in specific signaling pathways regulating peritoneal fibrosis. A recent study showed that intraperitoneal injection of 4.25% glucose PD solution could induce peritoneal thickening and activate β-catenin signal transduction in mice. Wnt/β-catenin signal plays a role in the progress of PD-related fibrosis by combining with TGF-β signal. (Guo et al., 2017). β-catenin has been reported to be cleaved by calpain in prostate cancer and breast cancer cells to maintain stability, and TCF-dependent transcriptional activity has increased. (Rios-Doria et al., 2004). Inhibition of calpain reduced TNF-α-induced β-catenin protein level. (Yi et al., 2020). *In vivo*, we found that the expression of β-catenin decreased after calpain was inhibited in the CG model with or without MDL28170, but no significant difference was observed in the PDF model. Previous studies showed that β-catenin in the cytoplasm of peritoneal mesothelial cells increased stability and continued accumulation under high glucose environment. (Kang, 2020). We observed that after calpain9 knockout of HmrSV5, β-catenin and FN expression decreased and e-cadherin expression increased, suggesting that calpain9 regulates one of the signal transduction molecules involved in EMT and TGF-β-induced fibrosis. We will explore the interaction of β-catenin with calpain in future studies to elucidate its possible mechanism.

In summary, in this study, we found that calpain was involved in peritoneal dialysis-related peritoneal fibrosis, and calpain9 may regulate peritoneal fibrosis through β-catenin, suggesting calpain9 may be a promising therapeutic target for the prevention and treatment of peritoneal fibrosis

## Data Availability

The raw data supporting the conclusion of this article will be made available by the authors, without undue reservation.
