# Supplementary figures and images for: Inhibition of calpain9 attenuates peritoneal dialysis-related peritoneal fibrosis
Source: Front Pharmacol. 2022 Dec 1;13:962770. doi: 10.3389/fphar.2022.962770 (PMC9751436; doi:10.3389/fphar.2022.962770)

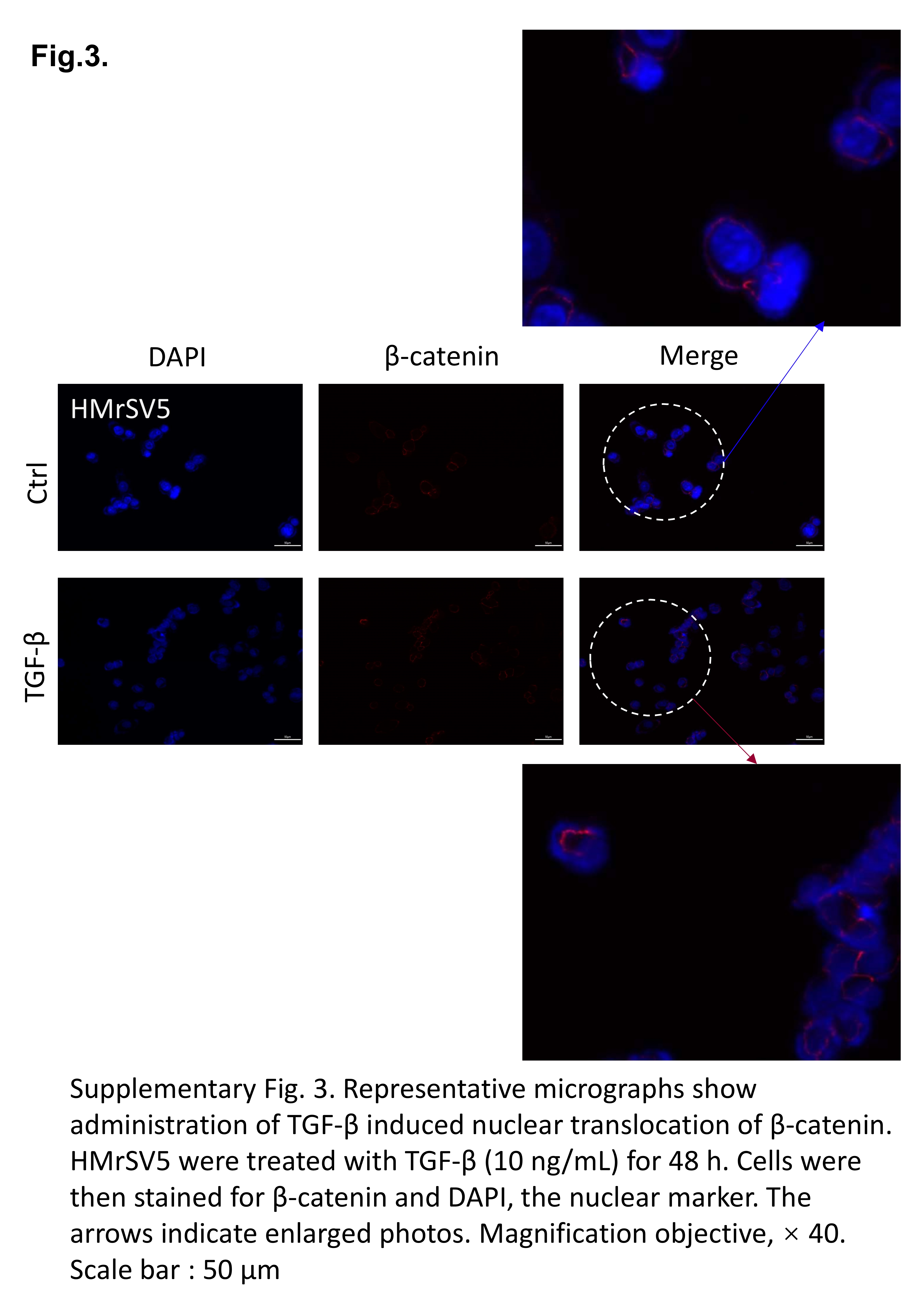

Supplement: Supplementary file 1 [file Image3.JPEG]

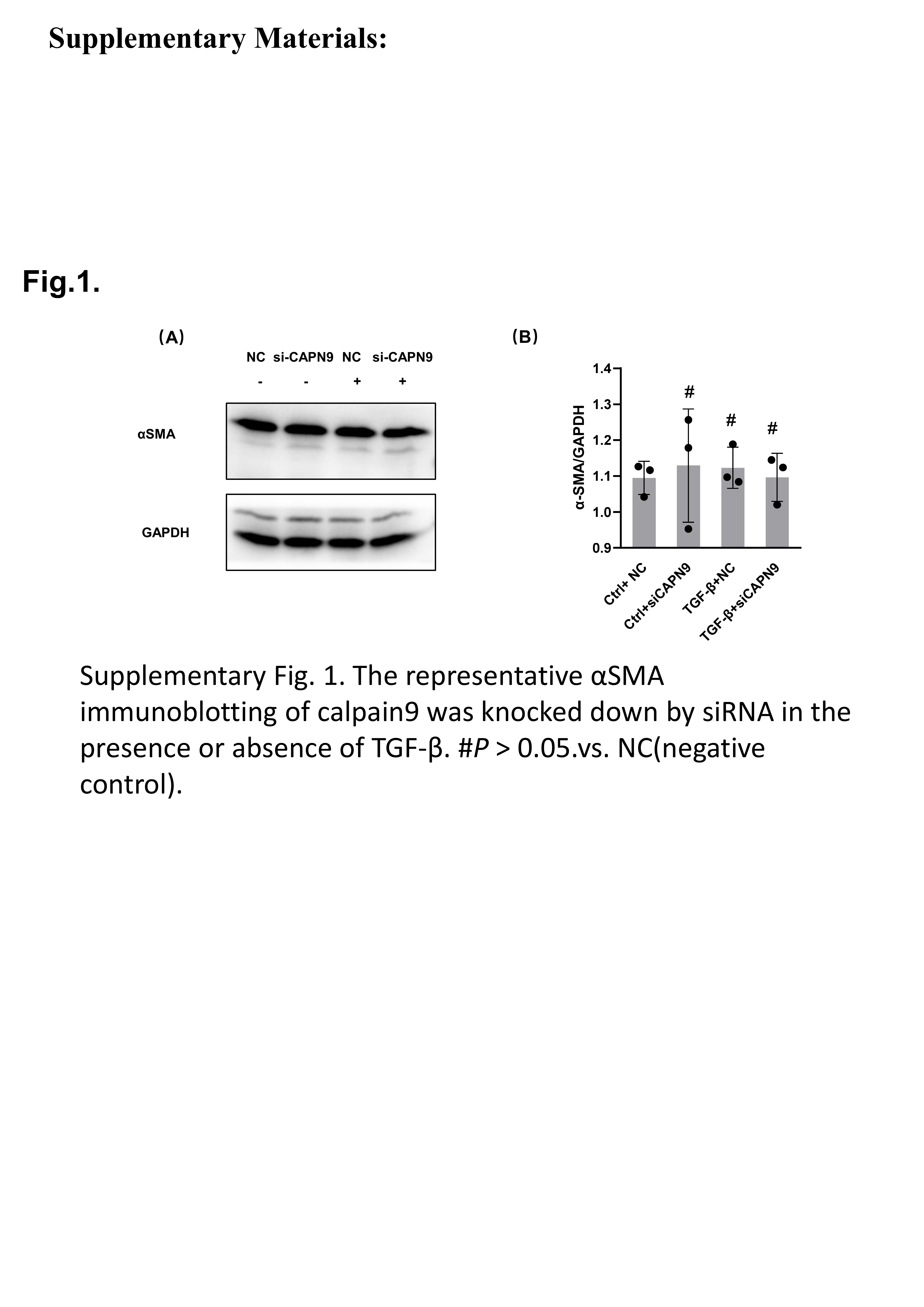

Supplement: Supplementary file 2 [file Image1.JPEG]

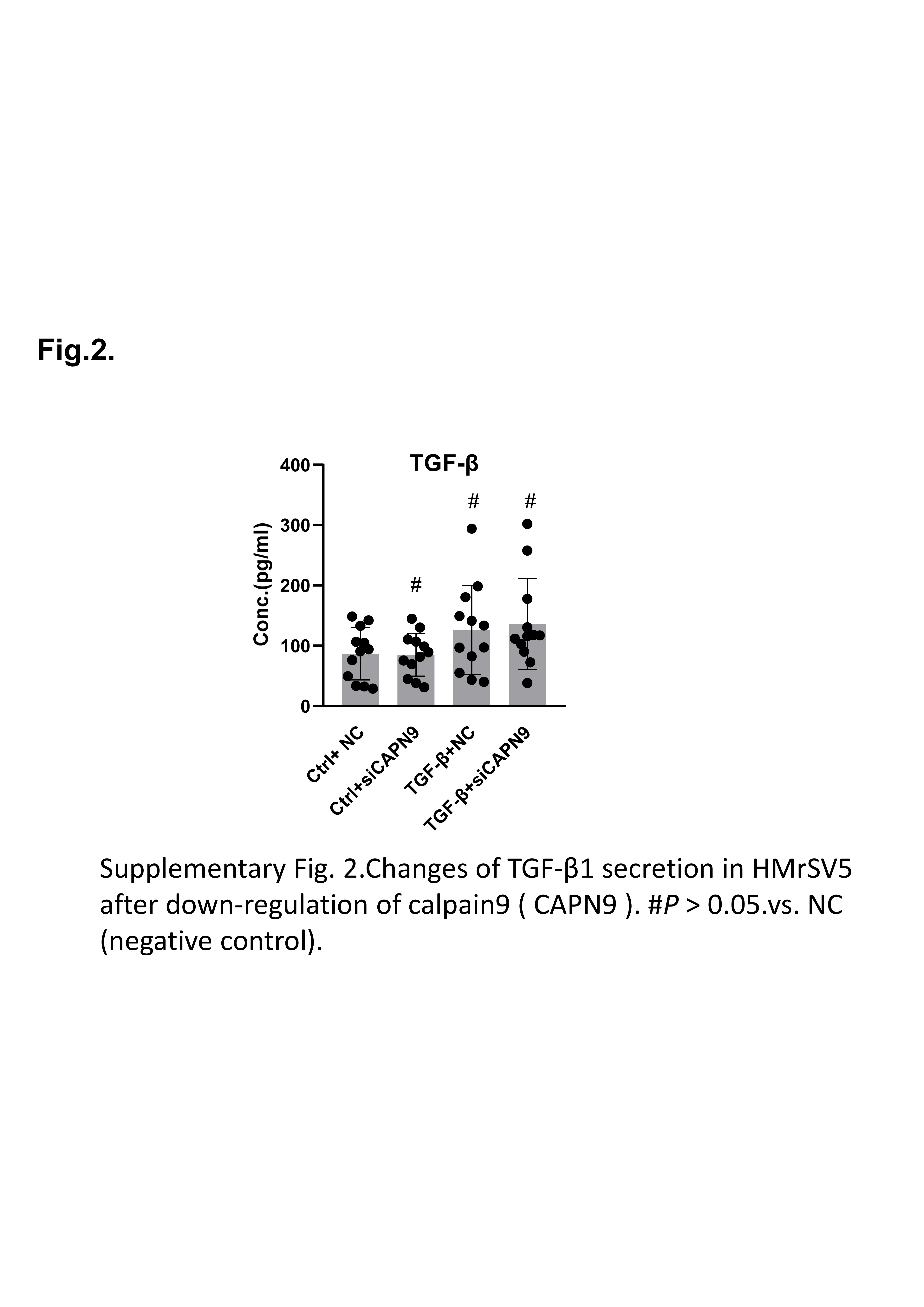

Supplement: Supplementary file 3 [file Image2.JPEG]
